# Lay People Esthetic Evaluation of Primary Surgical Repair on Three-Dimensional Images of Cleft Lip and Palate Patients

**DOI:** 10.3390/medicina55090576

**Published:** 2019-09-08

**Authors:** Edoardo Staderini, Marilisa De Luca, Ettore Candida, Maria Ida Rizzo, Oriana Rajabtork Zadeh, Daria Bucci, Mario Zama, Carlo Lajolo, Massimo Cordaro, Patrizia Gallenzi

**Affiliations:** 1 Institute of Dentistry and Maxillo-Facial Surgery, IRCCS Fondazione Policlinico “A. Gemelli”, Università Cattolica del Sacro Cuore, 00168 Rome, Italy (E.S.) (M.D.L.) (E.C.) (M.C.) (P.G.); 2 Craniofacial Centre-Plastic and Maxillofacial Surgery Unit of the Children’s Hospital Bambino Gesù, IRCCS, 00165 Rome, Italy (M.I.R.) (O.R.Z.) (D.B.) (M.Z.)

**Keywords:** cleft lip and palate, growth and development, reconstructive surgical procedures, therapy, soft tissue, treatment outcome, outcome assessment, photogrammetry

## Abstract

*Background and Objectives:* Previous literature has disclosed that facial attractiveness affects the esthetic evaluation of nose and lip deformity on frontal and lateral photographs. However, it has never been debated if the removal of the external facial features on three-dimensional (3D) models (“cropped assessment bias”) could provide a considerable usefulness in the interpretation and comparison of the results. Additionally, it has been assumed on two-dimensional (2D) studies that esthetic assessment biases with respect to observer gender, and it is not acknowledged if and to the extent that “gender assessment bias” may be influenced by a three-dimensional layout. The aim of this study is to investigate if facial traits and observers’ gender may affect the esthetic ratings of unilateral cleft lip and palate (UCLP) patients after soft tissue reconstruction. *Materials and Methods:* Three-dimensional images of ten UCLP patients’ images were acquired before the intervention (T0), one-month (T1) and six-months (T2) postoperative. Geomagic^®^ software (version 2014; 3D Systems, Rock Hill, SC, USA) was used to remove the external facial features of 3D surface models. Five-point scale developed by Asher-McDade et al. was used to rate both nasolabial attractiveness and impairment for full-face (FF) and cropped-face (CF) 3D images. Forty-three judges (21 males, 22 females) were enrolled for the esthetic evaluation. Intraclass correlation coefficient (ICC) was used to test intra- and inter-examiner reliability; a value of 0.7 was set as the minimum acceptable level of reliability. *Results:* When comparing the 2 sets of observations (FF and CF), the ICC ranged from 0.654 to 0.823. Concerning gender assessment bias, the ICC ranged from 0.438 to 0.686 and from 0.722 to 0.788 for males and females, respectively. Concerning inter-examiner reliability, ICC for questions 2–7 ranged from 0.448 to 0.644 and from 0.659 to 0.817 at T0 and T2, respectively. *Conclusions:* The removal of external facial features provides subtle differences on the esthetic assessment of UCLP patients. Moreover, based on our data, examiners’ gender differences may affect esthetic assessment of UCLP patients. Despite the subjectivity of esthetic judgments, a reliable, validated and reproducible scoring protocol should consider the influence of gender differences on 3D esthetic assessment of UCLP patients.

## 1. Introduction

### 1.1. Scientific Background

The management of cleft lip and palate (CLP) patients generally involves the soft palate reconstruction and the repair of lip and nasal soft tissue. Primary surgery accomplishes an improved facial appearance of CLP children as it guides maxillary segments towards a proper growth pattern and guarantees the development of a positive personality and self-esteem [1].

In order to avoid psychosocial consequences of cleft lip and palate from infancy to adulthood, it is important to rate the characteristic features of the nasolabial deformity and establish the need for further surgeries.

There is a lack of consensus regarding how CLP children are perceived by healthcare professionals, laypeople, caregivers and patient themselves [2,3].

Basically, knowledge instructs health professionals not to judge a cleft by the overall esthetic appearance of the face [4]. Therefore, lack of scientific background may lean layperson’ esthetic assessments towards a human natural inclination to judge people by their general facial attractiveness, thus misleading the perception of the nose and lip deformity [5].

It has been said that beauty is in the eye of the beholder; indeed, CLP perception may be influenced by the esthetic reference adopted by the evaluators. Observers with different backgrounds, race, gender, and age may tend to focus on characteristic features and trait impressions from people’s faces, comparing them to different stereotypes [6]. This concept implied caution against judging a cleft lip by the appearance of the confounding external facial features because it is supported by evidence that this does yield heterogeneous and subjective assessments [7].

Three-dimensional (3D) technology has also been raising its indications for assessment of facial appearance in CLP [8]. Advanced 3D imaging overcome standard two-dimensional (2D) limitations by visualizing patient’s face in all the directions and achieving a depth perception of the object—e.g., perspective, lighting, shading. Moreover, an innovative and interactive three-dimensional rendering attracts human attention and reduces the visual burden-to-fatigue [9].

Although there has been a rapid growth in 3D research, there is a need to develop clinical guidelines for facial aesthetic evaluation based on full-face (FF) or cropped-face (CF) 3D images [10].

### 1.2. Explanation of Rationale

The rationale of this clinical study is to investigate if facial attractiveness may influence the lay people assessment of pre-operative severity and post-operative esthetic outcome of patients with unilateral cleft lip and palate (UCLP).

The aim of this study is to examine whether the 3D images of faces of children with UCLP are inspected differently than faces of a control group with 3D display of isolated nasolabial areas.

### 1.3. Objective or Hypotheses

Therefore, a primary outcome and a secondary outcome are expected in this study.

- Primary outcome: How much the aesthetic judgment can be influenced by the complete view of the child’s face as compared to a cropped one, specifically related to the nose and lips (“cropped assessment bias”);
- Secondary outcome: How much the gender of the observer might affect the expression of laypeople judgment (“gender assessment bias”).

The null hypotheses were:

1. No difference exists between FF or CF 3D images;
2. Esthetic evaluation is not dependent on observers’ gender.

## 2. Materials and Methods

### 2.1. Trial Design

This clinical study (within-subject comparison) was realized at the Institute of Dentistry of “Fondazione Policlinico Agostino Gemelli” Hospital in cooperation with Craniofacial Centre of the Children’s Hospital “Bambino Gesù”; the study protocol was independently reviewed and approved by the Ethics Committee on 31 May 2017, with the ID number: 0027003/17. The present investigation was reported according to the CONSORT statement [11] (Supplementary File 1).

### 2.2. Baseline Data

Pediatric patients with a unilateral cleft lip and palate (UCLP) on the left or right side (7 males and 3 females) were prospectively recruited from the Craniofacial Centre of the “Bambino Gesù” Children’s Hospital (Rome, Italy), from 27 June 2017 to 22 September 2017.

The trial ended with the enrollment of UCLP patients; the sample enrolled was homogeneous for age, surgical technique, surgeon, treatment timing (Table 1).

Informed consent was obtained from all parents and legal guardians of the patients about the treatment plan, the participation to the study, and the use of 3D images of their children; the case study was conducted in compliance with the principles of the Declaration of Helsinki, and with the understanding and written consent of both parents (Supplementary File 2).

### 2.3. Data Collection

One day before the primary repair (T0), patients were transferred to Institute of Dentistry of University Cattolica del Sacro Cuore, where three-dimensional facial images were acquired. Each parent held the patient in his own arms to keep him in a natural head position with his eyes looking straight ahead to the camera. In order to catch infants’ attention, one operator (M.D.L.) behind the camera used bright or musical objects. Three different pictures were consecutively captured for each patient; one operator (E.C.) chose the best 3D acquisition, in terms of neutral facial expression and quality of the capture (eyes open, relaxed facial musculature, absence of head movements, Frankfort plane parallel to the floor, lips approximated lightly, no flattening of the chin) [12,13].

The 3D facial photographs were used with the same stereophotogrammetrical camera setup (3dMD face System; 3dMD LLC, Atlanta, GA, USA) by an experienced photographer. The 3dMD technology has been validated in terms of its accuracy and reliability [14]. The acquired images were loaded to 3dMDVultus editing software (3dMDpatient v.3.0.1, 3dMDVultusTM Software Platform, 3dMD LLC, Atlanta, GA, USA) and exported as 3D surface models [15].

The lip repair was performed at Bambino Gesù Children’s Hospital and follow-up visits have been set up one week, one-month and six-months after surgery. The 3D facial photographs were repeated for each child at one-month (T1) and six-month (T2) follow-up.

### 2.4. Data Preparation

**Figure 1:**
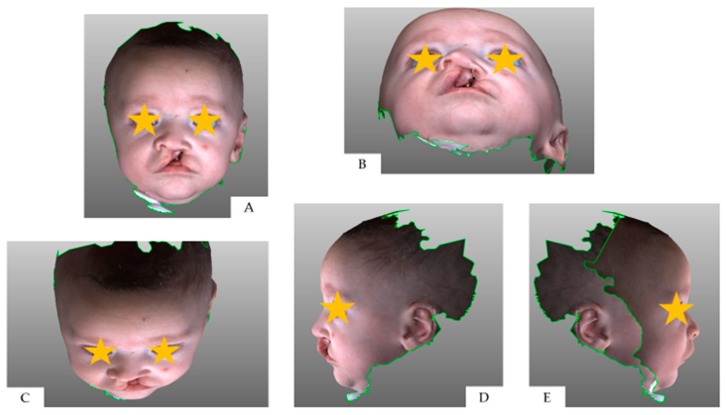
Three-dimensional full-face image at T0: (**A**) frontal view; (**B**) neck hyperflexion; (**C**) neck hyperflexion; (**D**) right profile; (**E**) left profile.

**Figure 2:**
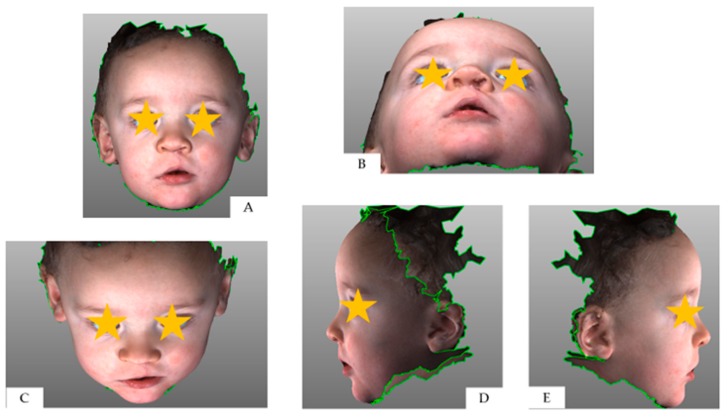
Three-dimensional full-face image at T2: (**A**) frontal view; (**B**) neck hyperflexion; (**C**) neck hyperflexion; (**D**) right profile; (**E**) left profile.

**Figure 3:**
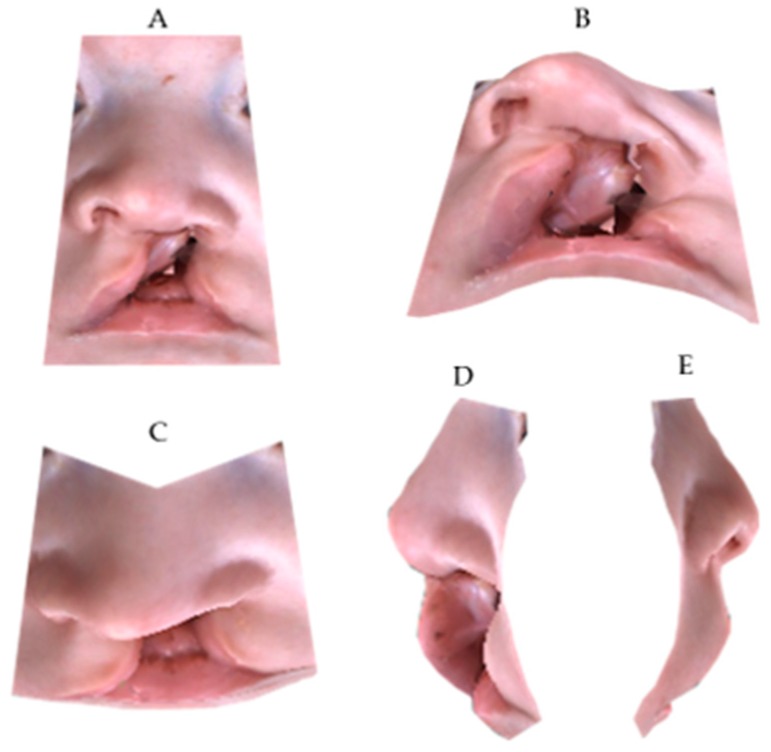
Three-dimensional cropped-face image at T0: (**A**) frontal view; (**B**) neck hyperflexion; (**C**) neck hyperflexion; (**D**) right profile; (**E**) left profile.

**Figure 4:**
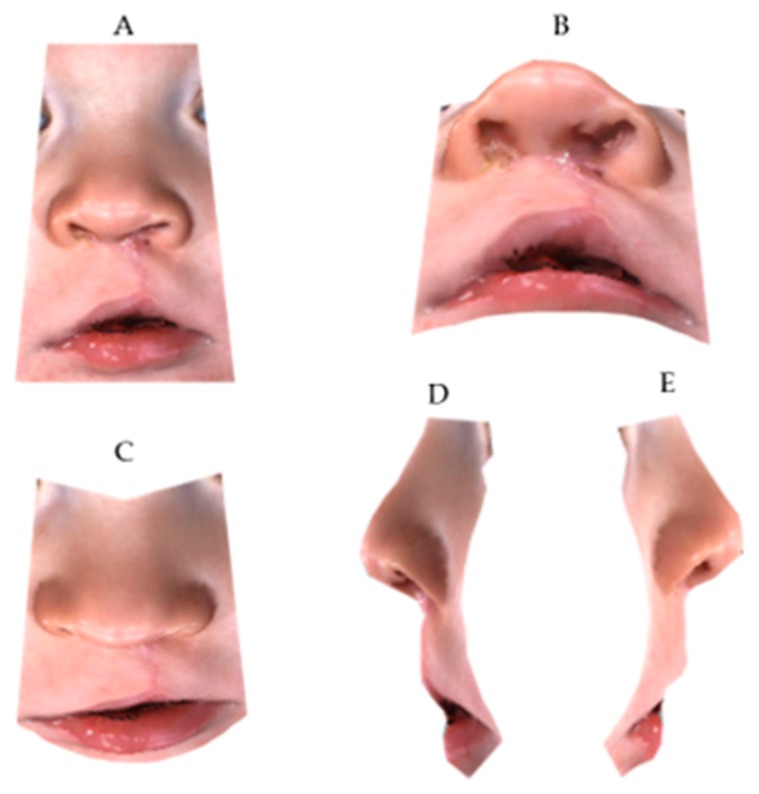
Three-dimensional cropped-face image at T2: (**A**) frontal view; (**B**) neck hyperflexion; (**C**) neck hyperflexion; (**D**) right profile; (**E**) left profile.

The Geomagic^®^ software (version 2014; 3D Systems, Rock Hill, SC, USA) was used to crop the 3D full-face models through a biphasic procedure. Points described by Desmedt et al. were selected on the 3D images in order to isolate the region of interest; indeed, the nasolabial area was confined within four planes passing through the identified anatomic landmarks [16] (Figure 1, Figure 2, Figure 3 and Figure 4).

Subsequently, the confounding external facial features were removed, and a cropped-face 3D model was obtained for each child.

### 2.5. Rating form Design

Subsequently, FF and CF 3D models were exported as “.obj” format; consecutive files display treatment progress of each child at T0, T1, and T2. In the rating form, full-face 3D images of all patients were presented before the cropped ones. FF and CF images were randomly arranged with a permuted blocks’ allocation generated by one investigator (E.S.) with http://randomization.com/. As a result, five different sequences of patients’ images were randomly generated and named with an identifying code.

### 2.6. Participants—Panel Sample Size and Composition

For this type of assessment, a preliminary sample size couldn’t be stated due to the methodological heterogeneity between the present investigation and current literature. Gkantidis et al. considered a minimum panel size of 12 judges to obtain a power of 80% with an alpha error minor to 0.05% [2]. Nevertheless, the authors conducted an aesthetic evaluation on two-dimensional images of adult patients; whereas to take account of a different study design, we set to enroll minimum number of 20 judges in each group to enlarge reliability of judgments. The lay panel selection was based on the checklist proposed by the systematic review of Bertens et al. [17] (Table 2).

### 2.7. Participants—Recruitment

Sixty-six volunteers were consecutively recruited by two investigators (M.C. and P.G.) as volunteers between January and April 2018; during the interview, an informed consent was obtained from the judges who agreed to take part in the study. Sixty-six laypeople were retrieved in the initial search; after the interview, fifty-eight met the inclusion/exclusion criteria; among them, seven people declined to participate, and two selected examiners didn’t join the rating session. Indeed, forty-nine judges took part at the evaluation process and forty-three rating forms were completed (Supplementary File 3).

### 2.8. Blinding

The judges were told in the explanatory phase that the study involved an aesthetic evaluation of “20 children” with cleft lip and palate: 10 patients provided with 3D full-face photographs, and then 10 patients with cropped 3D images of the nasolabial area. In order to avoid conditioning in their judgment, raters did not know that they were asked to analyze 3D nasolabial images trimmed from the FF pictures of the same patients.

### 2.9. Outcomes and Estimation—Selection of Assessment Scale

A brief summary was provided to the raters with a simple explanation of the rating form layout and the use of the Asher McDade scale for the aesthetic evaluation of the nasolabial area at T0, T1 and T2. In this current study, nine items of nasolabial morphology were assessed—nasal profile, labial profile, symmetry of the columella, nostril size, nostril shape, nostril symmetry, nose symmetry, wound healing, continuity of the vermilion border. The rating form was in Italian, and its English translation is available in Supplementary File 4. Asher McDade scale was accompanied by an explanatory legend; five possible scores were provided, namely: 1—very poor appearance; 2—poor appearance; 3—fair appearance; 4—good appearance; 5—very good appearance. The central score of 3 means that the facial appearance is intermediate value between the extremes of the scale; examiners were warned not to pick this option as an agreement with statements as presented (acquiescence bias) [18]. Each examiner was instructed to orientate FF and CF 3D model in a determined view (frontal, neck hyperflexion, neck hyperflexion, right and left profile) prior to answer to each question (Figure 1).

### 2.10. Evaluation Procedure

The rating process was biphasic: Firstly, the judges were asked to read an explanatory summary about the stereophotogrammetry, and the scoring system presented; any question addressed by the examiners was solved at this phase. Then, 3D sample images of UCLP patients were showed to the raters, who can orientate the face, familiarize with the 3D technology and have a first glance evaluation of the range of possible esthetic outcomes in UCLP condition. The 16 May 2018, judges were all gathered in a computer room with adequate lighting at University Cattolica del Sacro Cuore (Rome). Each examiner was instructed to cover the answer given before reading the next question in order to prevent this from affecting his/her next response. The judges were provided with an individual 15-inch laptop screen and asked to check the corresponding FF and CF 3D object projections; it was required to give one-shot answer, with no time limit for scoring. The overall rating form was compiled within 30 min.

### 2.11. Numbers Analysed

Drop-out of patients. All patients enrolled have completed the study; none of them has withdrawn.

Drop-out of judges. During the measurement, the judgments of two female and four male examiners were excluded because the rating form was partially compiled. A total of 43 rating forms (22 females, 21 males) were evaluated for primary and secondary outcome.

### 2.12. Statistical Methods

#### 2.12.1. Intra-Examiner Reliability—Primary Outcome

ICC test was used to determine the intra-examiner reliability for FF and CF ratings at time T0, T1, and T2.

#### 2.12.2. Intra-Examiner Reliability—Secondary Outcome

The same test was used to determine the intra-examiner reliability between male and female examiner scores at time T0, T1, and T2.

#### 2.12.3. Inter-Examiner Reliability

ICC test was used to determine the inter-examiner reliability for each question at time T0, T1, T2, evaluating separately CF and FF images. The ICC test was based both on each single question and on the overall score.

A coefficient major than 0.7 was considered reliable.

Data were reported on Excel (Microsoft Corp., Redmond, WA, USA) sheet. The analysis was performed using the statistical package SPSS v.25.0 (Chicago, IL, USA).

## 3. Results

### 3.1. Intra-Examiner Reliability—Primary Outcome

Excellent reliability was shown when comparing the 2 sets of observations (FF and CF) by the same examiners (Table 3).

### 3.2. Intra-Examiner Reliability—Secondary Outcome

The values of the female lay panel were higher than the corresponding male one. At one-month follow-up, the ratings of the female panel showed good reliability (>0.7), whereas poorly reliable values (<0.7) were found for the male panel (Table 4).

### 3.3. Inter-Examiner Reliability

The rating form reflects a consistent trend for CF and FF evaluations: questions 1, 8 and 9 showed good reliability (>0.7) in both groups (FF and CF) at all time periods. Meanwhile, questions from 2 to 7 were attributable to poorly reliable values (<0.7) at T0, but they showed good reliability at T1 and T2 (Table 5).

## 4. Discussion

The primary outcome of this study is to understand if three-dimensional images of children with unilateral cleft lip and palate need to be cropped before undergoing an esthetic assessment. The preparation of these images is burdened with methodological difficulties: the use of specific software to isolate the nasolabial area is time-consuming. We have not been able to find previous literature directly relating the influence of face cropping on the evaluation of three-dimensional images. Our findings demonstrate that the masking of surrounding facial features of 3D images of UCLP patients is not associated with significant modification of the Asher McDade scores. Even though, it is proving rather difficult to discover whether this difference is not due to confounding factors. Firstly, little differences between FF and CF images may be related to the short range of answers available on the 5-options scale. Secondly, the greater amount of healing tissue one month after surgery probably plays a substantial role during the esthetic evaluation of CF images. The healing tissue could exponentially draw the attention of the observer in CF images [19,20].

### 4.1. Interpretation in the Context of the Available Literature

Our findings are in agreement with those presented by Kocher et al., who analyzed the association between CF and FF images on two-dimensional images of adolescents. The authors conclude that the cropped modality of presentation cannot aid in esthetic assessment [21]. On the contrary, some studies have demonstrated how the eye area profoundly affects judgments on attractiveness and impairment [22]. However, in most studies, eye area is blurred because of privacy issues. Additionally, some methods of acquisition of three-dimensional images (e.g., laser scanner) require the patient’s eyes to be closed. For these considerations, we suggest to carefully eliminate the eye area in order to introduce a consistent scoring system for facial aesthetics in cleft patients. Several authors have reported significant differences in judgments related to the gender of the examiner [23,24,25]. Our study evidenced a greater consensus in women than in men between CF and FF images at 1-month postoperative assessment, thus confirming the relevant influence of the gender of the examiner on the assessment of three-dimensional images. Those findings may lay on psychological, social issues (i.e., satisfaction with appearance). However, based on our data, the greater or lesser criticality in relation to gender depends strongly on the question asked and it is not possible to indicate a single tendency for men and women. There is substantial evidence supporting the psychological and social traits of gender; the emphasis given to the physical appearance could influence the perception of people attractiveness [24]. Regarding the inter-examiner reliability, the presence of multiple anatomical structures could complicate the observers’ assessment of surgical outcomes.

### 4.2. Limitations and Strengths

The methodology of the present investigations has several strengths and may set important practical recommendations as yardsticks for lay panel assessment. Firstly, we focused on the esthetic assessment on UCLP babies, who exhibit structural deficiencies negatively affecting their facial appearance; indeed, emotional perception of a baby’s deformity should influence the judgment of primary repair of soft tissue defects. Regarding the study design, in the present investigation the judges were asked to:

- Answer consecutively, without any leaving blank answer, and successively, filling the rating form in the order proposed in the survey;
- Decide spontaneously; each examiner should ask himself/herself if patients who have unequal esthetics are differently rated in the rating form;
- Check each answer by visualizing the CF and FF 3D model.

Aims of the instructions were to minimize errors and bias occurring in esthetic scoring systems:

- Avoid that judges would discover that CF and FF images were derived from the same patients;
- Eliminate the tendency to avoid extreme response categories (central tendency bias);
- Avoid outguessing.

The proposed investigation also has weaknesses that must be acknowledged. First, the present investigation has involved the use of the Asher McDade scale; a five-points categorical aesthetic index could be useful for large epidemiological studies (Americleft, Eurocleft) due to their logistic and cost constraints, but it may distinguish less sensitively than VAS scale [26,27]. For these reasons, it could be useful to standardize the variability of ordinal scoring systems with appropriate verbal descriptors; furthermore, we partially minimize rater bias (central tendency bias, acquiescence bias) by selecting, training and monitoring each observer [28]. A weakness of the present study could be that the composition of the rating panel (lay people) may harm the generalizability of the results. As previously stated, a different pattern of responses could be found in different populations of judges. Previous studies showed that rating panels comprising laypersons were more critical, less critical, or equally critical to rating panels consisting of professionals (i.e., healthcare people involved in the treatment of cleft lip and palate). Indeed, the enrollment of different panels of judges may provide some challenging information regarding the influence of cultural background or ethno-racial differences on the assessment of facial appearance of UCLP children. A plausible disadvantage of this study could be the sample size of UCLP children involved in the esthetic evaluation; bilateral cleft lip and palate patients were not included because the numbers of patients affected was limited during the enrolment period. A study population of 10 patients may be considered relatively small; on the other hand, a long-lasting rating session could affect the burden-to-fatigue of the observers and increase the central tendency bias during the esthetic evaluation [29]. The aim of this study was to develop novel guidelines for 3D esthetic evaluation of the primary repair of UCLP patients. In view of the above considerations, an increase the UCLP sample size is not expected to enlarge generalizability of judgments.

## 5. Conclusions

Our findings demonstrate that, overall, 3D image cropping is not influential on the esthetic assessment of UCLP patients with Asher McDade scale. Our results provide a caveat to streamline the esthetic assessments and eliminate time-consuming procedures such as 3D image cropping. Although gender discrepancies in rating facial attractiveness might not have generalizability (facial attractiveness has not universal definition for lay people), a different consistency in the perception of surgical esthetic outcome was found in men and women [30]. Given those characteristics, a gender-matched cohort of judges is intended as primary criteria, whereas the surrounding facial feature does not fulfil a role as major confounding factor. In daily clinical practice, the use of lay panel matched for gender may involve some organizational efforts. On the other hand, the absence of the “data preparation” can largely compensate for the difficulties of the study design. Moreover, we aim to strengthen the importance of assessing UCLP patients with a 3D methodology that is not sensitive to a “flatness” in appearance that can exacerbate or mitigate judgments in scientific contexts. An evaluation of the impact of lip scarring would be helpful for investigating which methodological issues can potentially improve the 3D esthetic assessment of corrective surgeries.

## Figures and Tables

**Table 1:** Eligibility criteria for UCLP patients.

Criteria	Description
Age	Two-three months
Surgical technique	Millard’s repair
Surgeon	M.Z. ^1^
Treatment timing	Lip and nasal soft tissue repair at about 10 weeks of life
Health status	Absence of syndromic or severely compromised medical conditions for children
Medical history	Absence of previous orthopedic or surgical treatments on the oral district
Caregivers’ consensus	Decision to participate to the study, to submit the patient at surgical intervention and to follow multiple check-ups from birth onwards

^1^ M.Z.: Mario Zama.

**Table 2:** Eligibility criteria for lay panel.

Criteria	Description
Age	20–25 years
Gender	25 males and 25 females
Educational background	Undergraduate students without extensive professional knowledge on cleft lip and palate patients
Health status	Normal visual abilities without contact lenses, not using any medication that could affect vision or attentional control, absence of past or present neurological diseases.
Participation to similar studies	Absence of authorship or participation to similar research studies
Familiarity	Absence of familiarity or close friendship with UCLP ^1^ patients
Blindness	Blindness about aim of the research, study design and patient’s information.

^1^ UCLP: unilateral cleft lip and palate patients.

**Table 3:** Intra-examiner reliability between full-face and cropped-face 3D images.

	T0	T1	T2
Question 1	0.786 *	0.690	0.659
Question 2	0.798 *	0.728 *	0.756 *
Question 3	0.760 *	0.719 *	0.789 *
Question 4	0.781 *	0.686	0.817 *
Question 5	0.759 *	0.687	0.769 *
Question 6	0.776 *	0.732 *	0.812 *
Question 7	0.823 *	0.736 *	0.737 *
Question 8	-	0.706 *	0.791 *
Question 9	-	0.654	0.726 *

T0: Pre-operative; T1: one-month post-operative; T2: six-months post-operative. Question 1: nasal profile; Question 2: lip profile; Question 3: nostrils’ shape; Question 4: nostrils’ size; Question 5: symmetry of the columella; Question 6: nostrils’ symmetry; Question 7: nose symmetry; Question 8: wound healing; Question 9: continuity of the upper vermilion border. Questions 8 and 9 data are not available at T0, as they refer to the healing after surgery. *: reliability > 0.7.

**Table 4:** Intra-examiner reliability between CF and FF images for male and female ratings.

	Females			Males		
	T0	T1	T2	T0	T1	T2
Question 1	0.813 *	0.722 *	0.710 *	0.749 *	0.638	0.563
Question 2	0.820 *	0.757 *	0.800 *	0.772 *	0.678	0.652
Question 3	0.832 *	0.788 *	0.824 *	0.662	0.606	0.738 *
Question 4	0.836 *	0.762 *	0.845 *	0.721 *	0.552	0.773 *
Question 5	0.814 *	0.734 *	0.783 *	0.695	0.618	0.752 *
Question 6	0.823 *	0.788 *	0.826 *	0.720 *	0.646	0.792 *
Question 7	0.854 *	0.772 *	0.753 *	0.789 *	0.686	0.715 *
Question 8	-	0.731 *	0.794 *	-	0.670	0.786 *
Question 9	-	0.726 *	0.786 *	-	0.438	0.595

T0: Pre-operative; T1: one-month post-operative; T2: six-months post-operative. Question 1: nasal profile; Question 2: lip profile; Question 3: nostrils’ shape; Question 4: nostrils’ size; Question 5: symmetry of the columella; Question 6: nostrils’ symmetry; Question 7: nose symmetry; Question 8: wound healing; Question 9: continuity of the upper vermilion border. Questions 8 and 9 data are not available at T0, as they refer to the healing after surgery. *: reliability > 0.7.

**Table 5:** Inter-examiner reliability for FF and CF images.

	Full-Face			Cropped-Face		
	T0	T1	T2	T0	T1	T2
Question 1	0.824 *	0.849 *	0.855 *	0.831 *	0.872 *	0.885 *
Question 2	0.448	0.851 *	0.891 *	0.553	0.868 *	0.917 *
Question 3	0.632	0.789 *	0.884 *	0.502	0.853 *	0.880 *
Question 4	0.644	0.855 *	0.917 *	0.491	0.864 *	0.894 *
Question 5	0.461	0.829 *	0.833 *	0.476	0.812 *	0.827 *
Question 6	0.484	0.777 *	0.829 *	0.395	0.818 *	0.863 *
Question 7	0.639	0.803 *	0.833 *	0.598	0.813 *	0.864 *
Question 8	-	0.817 *	0.875 *	-	0.853 *	0.873 *
Question 9	-	0.892 *	0.890 *	-	0.907 *	0.891 *

T0: Pre-operative; T1: one-month post-operative; T2: six-months post-operative. Question 1: nasal profile; Question 2: lip profile; Question 3: nostrils’ shape; Question 4: nostrils’ size; Question 5: symmetry of the columella; Question 6: nostrils’ symmetry; Question 7: nose symmetry; Question 8: wound healing; Question 9: continuity of the upper vermilion border. Questions 8 and 9 data are not available at T0, as they refer to the healing after surgery. *: reliability > 0.7.

## Supplementary Materials

The following are available online at https://www.mdpi.com/1010-660X/55/9/576/s1, Supplementary file 1: CONSORT Checklist, Supplementary file 2: Informed consent, Supplementary file 3: CONSORT Flow Diagram, Supplementary file 4: Rating form for three-dimensional esthetic evaluation.

## References

[1] Zaleckas L., Linkeviciene L., Olekas J., Kutra N. (2011). The comparison of different surgical techniques used for repair of complete unilateral cleft lip. Medicina.

[2] Gkantidis N., Papamanou D.A., Christou P., Topouzelis N. (2013). Aesthetic outcome of cleft lip and palate treatment. Perceptions of patients, families, and health professionals compared to the general public. J. Cranio Maxillofac Surg..

[3] Molsted K., Humerinta K., Kuseler A., Skaare P., Bellardie H., Shaw W., Karsten A., Kare Saele P., Rizell S., Marcusson A. (2017). Scandcleft randomised trials of primary surgery for unilateral cleft lip and palate: 8. Assessing naso-labial appearance in 5-year-olds—A preliminary study. J. Plast. Surg. Hand Surg..

[4] Papamanou D.A., Gkantidis N., Topouzelis N., Christou P. (2012). Appreciation of cleft lip and palate treatment outcome by professionals and laypeople. Eur. J. Orthod..

[5] Paiva T.S., Andre M., Paiva W.S., Mattos B.S. (2014). Aesthetic evaluation of the nasolabial region in children with unilateral cleft lip and palate comparing expert versus nonexperience health professionals. BioMed Res. Int..

[6] Schwirtz R.M.F., Mulder F.J., Mosmuller D.G.M., Tan R.A., Maal T.J., Prahl C., de Vet H.C.W., Don Griot J.P.W. (2018). Rating nasolabial aesthetics in unilateral cleft lip and palate patients: Cropped versus full-face images. Cleft Palate Craniofac. J..

[7] Eichenberger M., Staudt C.B., Pandis N., Gnoinski W., Eliades T. (2014). Facial attractiveness of patients with unilateral cleft lip and palate and of controls assessed by laypersons and professionals. Eur. J. Orthod..

[8] Staderini E., Patini R., Camodeca A., Guglielmi F., Gallenzi P. (2019). Three-dimensional assessment of morphological changes following nasoalveolar molding therapy in cleft lip and palate patients: A case report. Dent. J..

[9] Shaw W.C., Rees G., Dawe M., Charles C.R. (1985). The influence of dentofacial appearance on the social attractiveness of young adults. Am. J. Orthod..

[10] Kuijpers M.A., Chiu Y.T., Nada R.M., Carels C.E., Fudalej P.S. (2014). Three-dimensional imaging methods for quantitative analysis of facial soft tissues and skeletal morphology in patients with orofacial clefts: A systematic review. PLoS ONE.

[11] Moher D. (1998). CONSORT: An evolving tool to help improve the quality of reports of randomized controlled trials. Consolidated Standards of Reporting Trials. JAMA.

[12] Saponaro G., Doneddu P., Gasparini G., Staderini E., Boniello R., Todaro M., D’Amato G., Pelo S., Moro A. (2019). Custom made onlay implants in peek in maxillofacial surgery: A volumetric study. Childs Nerv. Syst..

[13] Staderini E., Patini R., De Luca M., Gallenzi P. (2018). Three-dimensional stereophotogrammetric analysis of nasolabial soft tissue effects of rapid maxillary expansion: A systematic review of clinical trials. Acta Otorhinolaryngol. Ital..

[14] Heike C.L., Upson K., Stuhaug E., Weinberg S.M. (2010). 3D digital stereophotogrammetry: A practical guide to facial image acquisition. Head Face Med..

[15] Isidor S., Di Carlo G., Cornelis M.A., Isidor F., Cattaneo P.M. (2018). Three-dimensional evaluation of changes in upper airway volume in growing skeletal Class II patients following mandibular advancement treatment with functional orthopedic appliances. Angle Orthod..

[16] Desmedt D.J., Maal T.J., Kuijpers M.A., Bronkhorst E.M., Kuijpers-Jagtman A.M., Fudalej P.S. (2015). Nasolabial symmetry and esthetics in cleft lip and palate: Analysis of 3D facial images. Clin. Oral. Investig..

[17] Bertens L.C., Broekhuizen B.D., Naaktgeboren C.A., Rutten F.H., Hoes A.W., van Mourik Y., Moons K.G., Reitsma J.B. (2013). Use of expert panels to define the reference standard in diagnostic research: A systematic review of published methods and reporting. PLoS Med..

[18] Asher-McDade C., Roberts C., Shaw W.C., Gallager C. (1991). Development of a method for rating nasolabial appearance in patients with clefts of the lip and palate. Cleft Palate Craniofac. J..

[19] Tobiasen J.M., Hiebert J.M. (1993). Combined effects of severity of cleft impairment and facial attractiveness on social perception: An experimental study. Cleft Palate Craniofac. J..

[20] Ritter K., Trotman C.A., Phillips C. (2002). Validity of subjective evaluations for the assessment of lip scarring and impairment. Cleft Palate Craniofac. J..

[21] Kocher K., Kowalski P., Kolokitha O.E., Katsaros C., Fudalej P.S. (2016). Judgment of nasolabial esthetics in cleft lip and palate is not influenced by overall facial attractiveness. Cleft Palate Craniofac. J..

[22] Tobiasen J.M., Hiebert J.M., Boraz R.A. (1991). Development of scales of severity of facial cleft impairment. Cleft Palate Craniofac. J..

[23] Sinko K., Cede J., Jagsch R., Strohmayr A.L., McKay A., Mosgoeller W., Klug C. (2017). Facial aesthetics in young adults after cleft lip and palate treatment over five decades. Sci. Rep..

[24] Kiekens R.M., van ‘t Hof M.A., Straatman H., Kuijpers-Jagtman A.M., Maltha J.C. (2007). Influence of panel composition on aesthetic evaluation of adolescent faces. Eur. J. Orthod..

[25] Tobiasen J.M., Hiebert J.M. (1988). Reliability of esthetic ratings of cleft impairment. Cleft Palate J..

[26] Mercado A., Russell K., Hathaway R., Daskalogiannakis J., Sadek H., Long R.E., Cohen M., Semb G., Shaw W. (2011). The Americleft study: An inter-center study of treatment outcomes for patients with unilateral cleft lip and palate part 4. Nasolabial aesthetics. Cleft Palate Craniofac. J..

[27] Brattstrom V., Molsted K., Prahl-Andersen B., Semb G., Shaw W.C. (2005). The Eurocleft study: Intercenter study of treatment outcome in patients with complete cleft lip and palate. Part 2: Craniofacial form and nasolabial appearance. Cleft Palate Craniofac. J..

[28] Mosmuller D.G., Griot J.P., Bijnen C.L., Niessen F.B. (2013). Scoring systems of cleft-related facial deformities: A review of literature. Cleft Palate Craniofac. J..

[29] McLaughlin K., Ainslie M., Coderre S., Wright B., Violato C. (2009). The effect of differential rater function over time (DRIFT) on objective structured clinical examination ratings. Med. Educ..

[30] Al-Omari I., Millett D.T., Ayoub A.F. (2005). Methods of assessment of cleft-related facial deformity: A review. Cleft Palate Craniofac. J..

