# Supplementary material for: Lay People Esthetic Evaluation of Primary Surgical Repair on Three-Dimensional Images of Cleft Lip and Palate Patients
Source: Medicina (Kaunas). 2019 Sep 8;55(9):576. doi: 10.3390/medicina55090576 (PMC6780772; doi:10.3390/medicina55090576)
Supplement: Supplementary file 1 [file medicina-55-00576-s001.zip › Supplementary file 4.docx]

Date:

ESTHETIC EVALUATION IN PATIENTS WITH CLEFT LIP AND PALATE

Name of the examiner:

Baseline (pre-operative) esthetic assessment

1 - HOW DO YOU RATE THE APPEARANCE OF THE NASAL PROFILE (see profile views)?

1= VERY POOR APPEARANCE, 2 = POOR APPEARANCE, 3 = FAIR APPEARANCE

4= GOOD APPEARANCE, 5 = VERY GOOD APPEARANCE

2 - HOW DO YOU RATE THE APPEARANCE OF THE LIP PROFILE (see profile views)?

1= VERY POOR APPEARANCE, 2 = POOR APPEARANCE, 3 = FAIR APPEARANCE

4= GOOD APPEARANCE, 5 = VERY GOOD APPEARANCE

3 - HOW DO YOU RATE NOSTRILS’ SHAPE (see hyperextension and hyperflexion views)?

1= VERY POOR APPEARANCE, 2 = POOR APPEARANCE, 3 = FAIR APPEARANCE

4= GOOD APPEARANCE, 5 = VERY GOOD APPEARANCE

4 - HOW DO YOU RATE NOSTRILS’ SIZE (see hyperextension views)?

1= VERY POOR APPEARANCE, 2 = POOR APPEARANCE, 3 = FAIR APPEARANCE

4= GOOD APPEARANCE, 5 = VERY GOOD APPEARANCE

7 - HOW DO YOU RATE NOSE SIMMETRY (relative to an imaginary vertical midline between the inner canthi taken as midpoint - see frontal views)?

1= VERY POOR SIMMETRY, 2 = POOR SIMMETRY, 3 = FAIR SIMMETRY

4= GOOD SIMMETRY, 5 = VERY GOOD SIMMETRY

6 - HOW DO YOU RATE NOSTRILS’ SIMMETRY (see hyperextension views)?

1= VERY POOR SIMMETRY, 2 = POOR SIMMETRY, 3 = FAIR SIMMETRY

4= GOOD SIMMETRY, 5 = VERY GOOD SIMMETRY

5 - HOW DO YOU RATE SIMMETRY OF THE COLUMELLA (see hyperextension views)?

1= VERY POOR SIMMETRY, 2 = POOR SIMMETRY, 3 = FAIR SIMMETRY

4= GOOD SIMMETRY, 5 = VERY GOOD SIMMETRY

One-month post-operative esthetic assessment

1 - HOW DO YOU RATE THE APPEARANCE OF THE NASAL PROFILE (see profile views)?

1= VERY POOR APPEARANCE, 2 = POOR APPEARANCE, 3 = FAIR APPEARANCE

4= GOOD APPEARANCE, 5 = VERY GOOD APPEARANCE

2 - HOW DO YOU RATE THE APPEARANCE OF THE LIP PROFILE (see profile views)?

1= VERY POOR APPEARANCE, 2 = POOR APPEARANCE, 3 = FAIR APPEARANCE

4= GOOD APPEARANCE, 5 = VERY GOOD APPEARANCE

3 - HOW DO YOU RATE NOSTRILS’ SHAPE (see hyperextension and hyperflexion views)?

1= VERY POOR APPEARANCE, 2 = POOR APPEARANCE, 3 = FAIR APPEARANCE

4= GOOD APPEARANCE, 5 = VERY GOOD APPEARANCE

5 - HOW DO YOU RATE SIMMETRY OF THE COLUMELLA (see hyperextension views)?

1= VERY POOR SIMMETRY, 2 = POOR SIMMETRY, 3 = FAIR SIMMETRY

4= GOOD SIMMETRY, 5 = VERY GOOD SIMMETRY

4 - HOW DO YOU RATE NOSTRILS’ SIZE (see hyperextension views)?

1= VERY POOR APPEARANCE, 2 = POOR APPEARANCE, 3 = FAIR APPEARANCE

4= GOOD APPEARANCE, 5 = VERY GOOD APPEARANCE

9 - HOW DO YOU RATE WOUND HEALING (see frontal views)?

1= VERY POOR APPEARANCE, 2 = POOR APPEARANCE, 3 = FAIR APPEARANCE

4= GOOD APPEARANCE, 5 = VERY GOOD APPEARANCE

8 - HOW DO YOU RATE CONTINUITY OF THE UPPER VERMILION BORDER (see frontal views)?

1= VERY POOR APPEARANCE, 2 = POOR APPEARANCE, 3 = FAIR APPEARANCE

4= GOOD APPEARANCE, 5 = VERY GOOD APPEARANCE

7 - HOW DO YOU RATE NOSE SIMMETRY (relative to an imaginary vertical midline between the inner canthi taken as midpoint - see frontal views)?

1= VERY POOR SIMMETRY, 2 = POOR SIMMETRY, 3 = FAIR SIMMETRY

4= GOOD SIMMETRY, 5 = VERY GOOD SIMMETRY

6 - HOW DO YOU RATE NOSTRILS’ SIMMETRY (see hyperextension views)?

1= VERY POOR SIMMETRY, 2 = POOR SIMMETRY, 3 = FAIR SIMMETRY

4= GOOD SIMMETRY, 5 = VERY GOOD SIMMETRY

Six-months post-operative esthetic assessment

1 - HOW DO YOU RATE THE APPEARANCE OF THE NASAL PROFILE (see profile views)?

1= VERY POOR APPEARANCE, 2 = POOR APPEARANCE, 3 = FAIR APPEARANCE

4= GOOD APPEARANCE, 5 = VERY GOOD APPEARANCE

2 - HOW DO YOU RATE THE APPEARANCE OF THE LIP PROFILE (see profile views)?

1= VERY POOR APPEARANCE, 2 = POOR APPEARANCE, 3 = FAIR APPEARANCE

4= GOOD APPEARANCE, 5 = VERY GOOD APPEARANCE

3 - HOW DO YOU RATE NOSTRILS’ SHAPE (see hyperextension and hyperflexion views)?

1= VERY POOR APPEARANCE, 2 = POOR APPEARANCE, 3 = FAIR APPEARANCE

4= GOOD APPEARANCE, 5 = VERY GOOD APPEARANCE

5 - HOW DO YOU RATE SIMMETRY OF THE COLUMELLA (see hyperextension views)?

1= VERY POOR SIMMETRY, 2 = POOR SIMMETRY, 3 = FAIR SIMMETRY

4= GOOD SIMMETRY, 5 = VERY GOOD SIMMETRY

4 - HOW DO YOU RATE NOSTRILS’ SIZE (see hyperextension views)?

1= VERY POOR APPEARANCE, 2 = POOR APPEARANCE, 3 = FAIR APPEARANCE

4= GOOD APPEARANCE, 5 = VERY GOOD APPEARANCE

9 - HOW DO YOU RATE WOUND HEALING (see frontal views)?

1= VERY POOR APPEARANCE, 2 = POOR APPEARANCE, 3 = FAIR APPEARANCE

4= GOOD APPEARANCE, 5 = VERY GOOD APPEARANCE

8 - HOW DO YOU RATE CONTINUITY OF THE UPPER VERMILION BORDER (see frontal views)?

1= VERY POOR APPEARANCE, 2 = POOR APPEARANCE, 3 = FAIR APPEARANCE

4= GOOD APPEARANCE, 5 = VERY GOOD APPEARANCE

7 - HOW DO YOU RATE NOSE SIMMETRY (relative to an imaginary vertical midline between the inner canthi taken as midpoint - see frontal views)?

1= VERY POOR SIMMETRY, 2 = POOR SIMMETRY, 3 = FAIR SIMMETRY

4= GOOD SIMMETRY, 5 = VERY GOOD SIMMETRY

6 - HOW DO YOU RATE NOSTRILS’ SIMMETRY (see hyperextension views)?

1= VERY POOR SIMMETRY, 2 = POOR SIMMETRY, 3 = FAIR SIMMETRY

4= GOOD SIMMETRY, 5 = VERY GOOD SIMMETRY
