# Supplementary figures and images for: Lay People Esthetic Evaluation of Primary Surgical Repair on Three-Dimensional Images of Cleft Lip and Palate Patients
Source: Medicina (Kaunas). 2019 Sep 8;55(9):576. doi: 10.3390/medicina55090576 (PMC6780772; doi:10.3390/medicina55090576)

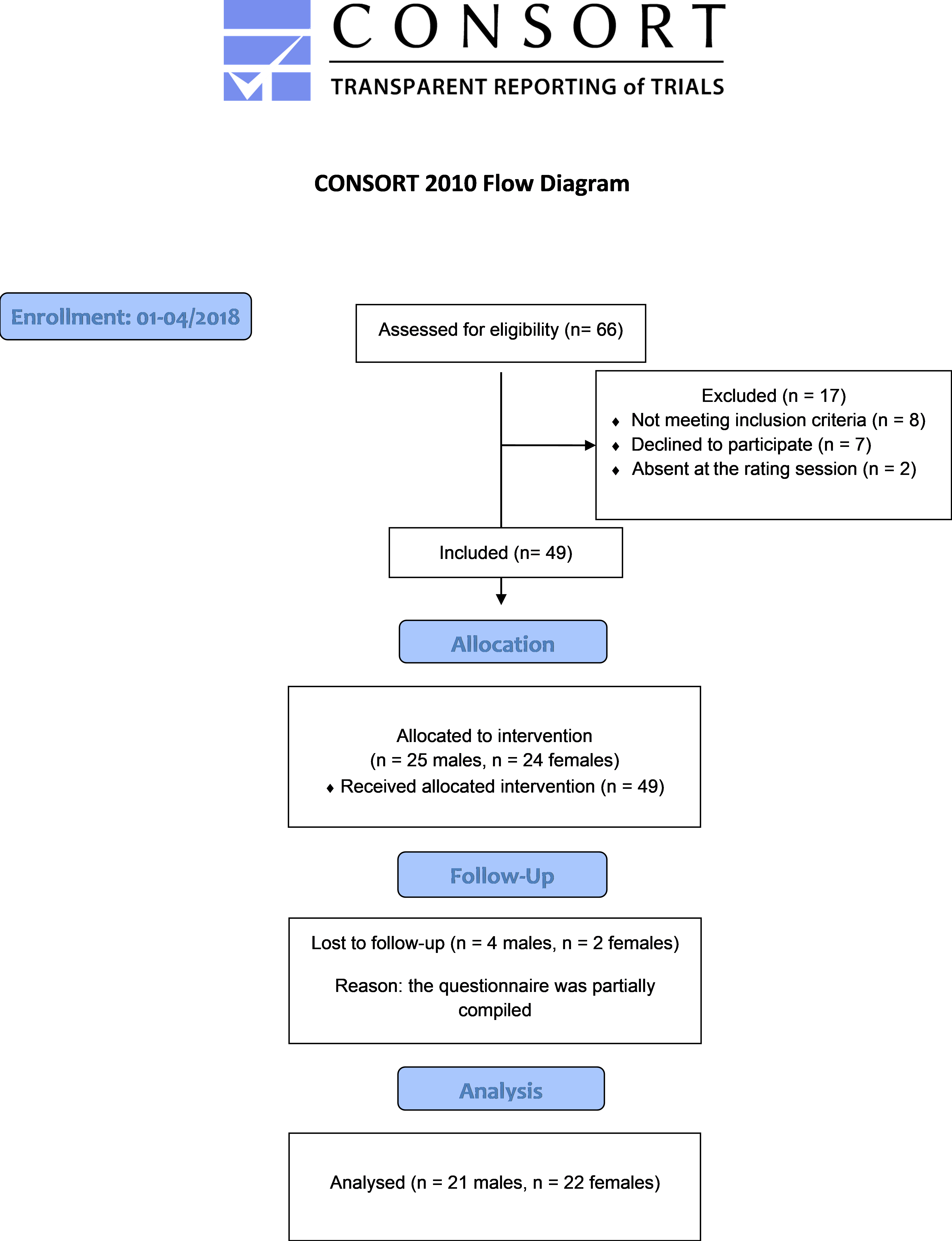

Supplement: Supplementary file 1 [file medicina-55-00576-s001.zip › Supplementary file 3.tif]
