# Supplementary material for: Lay People Esthetic Evaluation of Primary Surgical Repair on Three-Dimensional Images of Cleft Lip and Palate Patients
Source: Medicina (Kaunas). 2019 Sep 8;55(9):576. doi: 10.3390/medicina55090576 (PMC6780772; doi:10.3390/medicina55090576)
Supplement: Supplementary file 1 [file medicina-55-00576-s001.zip › Supplementary file 2.doc]

**Informativa per il trattamento dei dati per scopi di sperimentazione clinica per gli studi no profit**

*(Art. 13 del D.Lgs. 196/2003 ”Codice in materia di protezione dei dati personali”)*

**CONSENSO PER IL TRATTAMENTO DEI DATI PERSONALI**

**IN CASO DI STUDI NO PROFIT**

*Versione n. 1 del 9/02/2017*

**TITOLO DELLO STUDIO:** *Efficacia del trattamento chirurgico maxillo-facciale sul volto dei bambini con labiopalatoschisi: valutazioni estetiche ed ortodontiche*

Ai sensi del D.Lgs. 30 giugno 2003, n.196 ”Codice in materia di protezione dei dati personali” (di seguito “Codice”), la **Fondazione Policlinico Universitario Agostino Gemelli** (nel seguito per brevità anche “***Fondazione***”) **con sede in Largo Francesco Vito, n. 1 – 00168 – Roma**, in qualità di Titolare del trattamento, La informa che i Suoi dati, acquisiti attraverso il modulo allegato, saranno trattati secondo le modalità di seguito riportate.

**1. FINALITÀ DEL TRATTAMENTO E NATURA DEI DATI**

Il presente trattamento, avente ad oggetto i Suoi dati *personali* (qualsiasi informazione relativa alla persona fisica ivi compreso un numero di identificazione personale), *sensibili* (ad es. quelli relativi allo stato di salute) ed eventualmente *genetici* (dati, che riguardano i caratteri ereditari di un individuo), verrà effettuato per finalità di:

1. sperimentazione clinica, secondo quanto disposto dal D.Lgs. 196/2003, da leggi e regolamenti nazionali ed internazionali, dai Codici di deontologia e di buona condotta emanati in ambito medico e sanitario, nonché dai Provvedimenti emanati dall’Autorità Garante in materia;
2. ricerca scientifica e statistica finalizzata alla tutela della salute della collettività in campo medico, biomedico ed epidemiologico;
3. tutela della salute con particolare riferimento alle patologie di natura genetica ed alla tutela dell’identità;
4. adempimento di specifici obblighi o esecuzione di specifici compiti previsti espressamente dalla normativa internazionale e comunitaria, nonché da leggi o da regolamenti vigenti in materia.

**2. NATURA DEL CONFERIMENTO DEI DATI**

Il conferimento dei Suoi dati è necessario per il conseguimento delle finalità sopra richiamate.

La partecipazione alla sperimentazione avviene su base volontaria, pertanto, il mancato consenso al trattamento dei dati per tale finalità non pregiudica il diritto di avvalersi delle altre prestazioni medico sanitarie erogate dalla Fondazione.

**3. MODALITÀ DI TRATTAMENTO DEI DATI**

Tali finalità prevedono lo svolgimento delle operazioni di raccolta, registrazione, conservazione e modificazione dei dati personali mediante strumenti manuali ed informatici con logiche strettamente correlate alle finalità stesse e, comunque, in modo da garantire la sicurezza e la riservatezza dei dati stessi.

I soggetti incaricati del trattamento dei dati, porranno specifica attenzione nel differenziare i dati raccolti a scopo medico/clinico da quelli raccolti per finalità di sperimentazione nel rispetto della tutela dei diritti e della dignità del paziente.

I Suoi dati potranno essere trattati con la collaborazione di soggetti terzi espressamente nominati dal Titolare, Responsabili o Incaricati del Trattamento.

**4. COMUNICAZIONE E DIFFUSIONE**

I Suoi dati potranno essere comunicati a terzi nei casi in cui le strutture e le dotazioni interne della Fondazione risultassero inadeguate al perseguimento delle finalità oggetto del trattamento o nei casi espressamente previsti da disposizione di legge o regolamento.

Il Comitato etico e le autorità sanitarie italiane e straniere, potranno conoscere i dati che La riguardano, contenuti anche nella Sua documentazione clinica originale con modalità tali da garantire la riservatezza della Sua identità.

Qualora risulti necessario, i Suoi dati potranno essere trasmessi in paesi terzi appartenenti o non appartenenti all’Unione Europea, i quali potrebbero non garantire un adeguato livello di protezione dei dati (ad es. Stati Uniti); la diffusione dei Suoi dati, al di fuori dei casi citati, avverrà esclusivamente in forma anonima.

I dati che emergeranno dai risultati delle analisi, anche quelle future, qualora comportino un beneficio concreto e diretto in termini di terapia, prevenzione o di consapevolezza delle Sue scelte future, saranno resi noti direttamente a Lei; solamente con il Suo consenso potranno essere resi noti a persone diverse, da Lei specificatamente indicate.

**5. CONSERVAZIONE**

I dati da Lei forniti saranno conservati per un arco di tempo non superiore a quello necessario per conseguire le finalità per le quali sono stati raccolti e trattati.

In particolar modo, i dati emergenti da sperimentazione saranno conservati nei limiti stabiliti dalle leggi che regolano la materia.

**6. ESERCIZIO DEI DIRITTI**

Le comunichiamo inoltre che agli effetti della Legge, Titolare del trattamento è la Fondazione Policlinico Universitario Agostino Gemelli, con sede in Largo Francesco Vito n. 1 – 00168 – Roma.

Ai sensi dell’art. 7 del Codice, Lei potrà ottenere la conferma dell’esistenza o meno dei medesimi dati, di conoscerne il contenuto e l’origine, verificarne l’esattezza o chiederne l’integrazione o l’aggiornamento, oppure la rettificazione nonché conoscere l’elenco aggiornato dei Responsabili del Trattamento, con una comunicazione al titolare del trattamento dei dati attraverso la Segreteria del Comitato Etico. Potrà inoltre interrompere in ogni momento e senza fornire alcuna giustificazione la Sua partecipazione alla sperimentazione; in tal caso non saranno più raccolti ulteriori dati che la riguardano, ferma restando l’utilizzazione di quelli eventualmente già raccolti per determinare, senza alterarli, i risultati della ricerca.

In caso di interruzione del trattamento, i campioni biologici eventualmente prelevati saranno distrutti.

**Consenso al trattamento dei dati personali**

***ai sensi dell’art. 23 del D.Lgs. 30 giugno 2003, n. 196***

**Consenso al trattamento dei dati per scopi di sperimentazione**

Preso atto dell’informativa di cui all’art. 13 del D.Lgs. 196/2003, il sottoscritto______________________, nato a_________________, il__________, residente in_______ (Provincia ______), Via _________, N°_____, CAP _________, tel. _________________________

in qualità di esercente la potestà di genitore del minore (*è necessario il consenso congiunto di entrambi i genitori*)

Padre ____________________, nato a___________, il ____________, residente__________________

Madre ____________________, nata a___________, il ____________, residente__________________

*Oppure*

- amministratore di sostegno dell’incapace naturale/rappresentante legale__________________, nato a______________, il __________, residente__________________

□ **dà il proprio consenso**	□ **nega il proprio consenso**

al trattamento dei dati per finalità relative alla sperimentazione clinica

□ **dà il proprio consenso**	□ **nega il proprio consenso**

al trattamento dei dati ed al loro trasferimento in paesi terzi appartenenti o non appartenenti all'Unione europea per scopi di sperimentazione/ricerca nei limiti e con le modalità indicate nell'informativa fornita con il presente documento

□ **dà il proprio consenso**	□ **nega il proprio consenso**

alla eventuale cessione dei dati in forma anonima ad aziende farmaceutiche o ad altri soggetti che utilizzino gli stessi a scopo di studio o ricerca

□ **dà il proprio consenso**	□ **nega il proprio consenso**

affinché i risultati delle analisi e di eventuali scoperte inattese che emergano durante le attività di sperimentazione siano comunicate a:

□ me medesimo

□ familiare (Cognome e nome _________________)

□ convivente /coniuge (Cognome e nome _________________)

□ medico di famiglia (Cognome e nome _________________)

**Sezione da compilare nel caso in cui la sperimentazione tratti dati genetici**

Nel caso in cui gli esiti dei *test,* nonché i risultati delle ricerche, comportino un beneficio concreto e diretto in termini di terapia, prevenzione o di consapevolezza nelle scelte riproduttive

**DICHIARA**

□ di voler conoscere tali informazioni	□ di non voler conoscere tali informazioni (*solo nel caso la mancata informazione non sia di pericolo per sé o per gli altri)*

Nel caso in cui gli esiti dei *test,* nonché i risultati delle ricerche, comportino un beneficio concreto e diretto in termini di terapia, prevenzione o di consapevolezza delle scelte riproduttive, anche per gli appartenenti alla stessa linea genetica

**DICHIARA**

□ di acconsentire alla comunicazione di tali informazioni a questi ultimi, qualora ne facciano richiesta

□ di non acconsentire alla comunicazione di tali informazioni a questi ultimi, qualora ne facciano richiesta

Nel caso in cui la Fondazione non effettui, presso i propri laboratori, l’analisi richiesta

**DICHIARA**

□ di acconsentire all’invio dei campioni biologici presso il Laboratorio di ___________________ , esterno alla Fondazione Policlinico Universitario Agostino Gemelli, per l’esecuzione del seguente esame ________________________

□ di non acconsentire al trasferimento dei campioni biologici presso il Laboratorio di ________________ , esterno alla Fondazione Policlinico Universitario Agostino Gemelli, per l’esecuzione del seguente esame ________________________.

Roma, lì __________________________________	Firma del dichiarante __________________
